# Comparison of pulmonary and extrapulmonary tuberculosis in Nepal- a hospital-based retrospective study

**DOI:** 10.1186/1471-2334-8-8

**Published:** 2008-01-24

**Authors:** Chandrashekhar T Sreeramareddy, Kishore V Panduru, Sharat C Verma, Hari S Joshi, Michael N Bates

**Affiliations:** 1 Department of Community Medicine, Manipal Teaching Hospital, Manipal College of Medical Sciences, Pokhara, Nepal; 2 Department of Internal Medicine, Manipal Teaching Hospital, Manipal College of Medical Sciences, Pokhara, Nepal; 3 Director and Senior Chest Physician, Regional Tuberculosis Centre, Pokhara, Nepal; 4 School of Public Health, University of California, Berkeley, CA 94720, USA

## Abstract

**Background:**

Studies from developed countries have reported on host-related risk factors for extra-pulmonary tuberculosis (EPTB). However, similar studies from high-burden countries like Nepal are lacking. Therefore, we carried out this study to compare demographic, life-style and clinical characteristics between EPTB and PTB patients.

**Methods:**

A retrospective analysis was carried out on 474 Tuberculosis (TB) patients diagnosed in a tertiary care hospital in western Nepal. Characteristics of demography, life-style and clinical features were obtained from medical case records. Risk factors for being an EPTB patient relative to a PTB patient were identified using logistic regression analysis.

**Results:**

The age distribution of the TB patients had a bimodal distribution. The male to female ratio for PTB was 2.29. EPTB was more common at younger ages (< 25 years) and in females. Common sites for EPTB were lymph nodes (42.6%) and peritoneum and/or intestines (14.8%). By logistic regression analysis, age less than 25 years (OR 2.11 95% CI 1.12–3.68) and female gender (OR 1.69, 95% CI 1.12–2.56) were associated with EPTB. Smoking, use of immunosuppressive drugs/steroids, diabetes and past history of TB were more likely to be associated with PTB.

**Conclusion:**

Results suggest that younger age and female gender may be independent risk factors for EPTB in a high-burden country like Nepal. TB control programmes may target young and female populations for EPTB case-finding. Further studies are necessary in other high-burden countries to confirm our findings.

## Background

Tuberculosis (TB) remains a major global public health problem [1]. It is estimated that about one-third of the world's population is infected with *mycobacterium tuberculosis *[2]. After primary infection, TB may reactivate at anytime and anywhere in the body. Recent studies have suggested that the sites of extra-pulmonary tuberculosis (EPTB) may vary according to geographic location and population [3-6]. Clinical manifestations of TB are variable and depend on a number of factors that are related to the microbe, the host and the environment [7]. Our understanding of the role of host-related factors responsible for the occurrence of TB at extra-pulmonary sites is limited. Some studies have reported that the proportion of TB that is EPTB is on the rise due to the HIV epidemic [8,9] and possibly also improvement in diagnostic facilities [10]. Some studies have examined the role of host-related factors on the risk of development of EPTB. A study from the USA, reported that women, non-Hispanic blacks and HIV-infected individuals were at higher risk of EPTB [3]. Another US study reported that being African American, HIV-seropositive, less than 18 years of age and having liver cirrhosis were risk factors for EPTB [11]. A study from Turkey reported that females had a higher risk of developing EPTB and the risk of EPTB increased five years after initial contact [5]. One study reported that host-related factors for EPTB varied according to geographic origin and risk factors for EPTB were female gender for individuals of Asian or North African origin, age for individuals who originated from sub Saharan Africa and HIV infection for Europeans [12].

Nepal is a high-burden country for TB. About 45% of the total population is infected with TB and an estimated 20,000 new infectious cases of TB are reported each year [13]. The estimated annual incidence rate for all types of TB disease is 187 per 100,000 population. The annual incidence of new smear-positive TB cases is 83.5 per 100,000 population [14]. Sentinel surveys have reported that the prevalence of HIV seropositivity among TB patients has increased from 0.6% in 1995/96 to 2.4% in 2001/02 [13]. Risk factors for EPTB in Nepal may be different to those in low-burden countries, but appropriate studies to investigate this are lacking. We carried out this study to identify possible risk factors for EPTB that are distinctive from risk factors for PTB.

## Methods

### Study setting

Manipal Teaching Hospital (MTH) is a tertiary care hospital affiliated to Manipal College of Medical Sciences (MCOMS) serving the population of Pokhara city and remote hilly areas of western Nepal. The total population served by the hospital is about 380000. In March 2003, a TB treatment (DOTS) clinic was started in MTH, operating under the National Tuberculosis Program (NTP) of Nepal, under which the diagnosis of pulmonary TB is followed by examination of three sputum smears by Ziehl Nielssen staining for acid fast bacilli [AFB]. Chest radiographs are also used to support the diagnosis. At MTH, patients diagnosed with TB are referred to the DOTS clinic where they are registered and treated according to NTP guidelines [13].

### Data collection

A total of 526 TB patients registered in the DOTS clinic from March 31, 2003 to May 30, 2006 were included in the study. Patients' registration numbers were used to obtain corresponding files from the medical records department. From each medical case file, the patient's history, physical findings, chest radiographs and reports of laboratory investigations were reviewed to obtain the necessary information about diagnosis of TB. For each patient, demographic information, lifestyle factors (smoking habits and alcohol use) and clinical characteristics were recorded. Clinical characteristics included were, co-morbid conditions (diabetes mellitus, malignancies etc), treatment history (use of immunosuppressive drugs, steroids), past history of treatment for TB, history of known contact with a case of TB and the interval between contact with a TB patient and onset of the disease.

### Classification of PTB and EPTB patients

It is well known that lymph node and pleural involvement in TB is a direct extension of the disease from lung parenchyma. Therefore, patients with exclusively intrathoracic involvement (i.e., confined to lung parenchyma, pleura, and intrathoracic lymph nodes) were considered as PTB for the purpose of this analysis. Patients with extension of disease to organs or tissues outside the thorax, including those patients who also had pulmonary involvement, were considered as EPTB in our analysis. We followed the definitions used in two earlier studies [3,5] for EPTB and PTB patients.

The following clinical case definitions as recommended by the World Health Organization [15], after some modifications were used:

#### Pulmonary tuberculosis

Two or three initial sputum smear examinations positive for AFB, or one sputum smear positive for AFB plus radiographic abnormalities consistent with active pulmonary tuberculosis, as determined by a clinician, or one sputum smear positive for AFB (smear-positive cases). Cases with three sputum smears negative for AFB but clinical and radiological features compatible with active tuberculosis and showing improvement after empirical anti-tuberculosis treatment were considered to be smear-negative cases. Both smear-positive and smear-negative patients were treated as pulmonary TB cases for our data analysis.

#### Extra-pulmonary TB

This included tuberculosis of organs other than the lungs, such as lymph nodes, abdomen, genitourinary tract, skin, joints and bones, meninges, etc. Diagnosis of EPTB was based on fine needle aspiration cytology or biochemical analyses of cerebrospinal/pleural/ascitic fluid or histopathological examination or strong clinical evidence consistent with active extra-pulmonary tuberculosis, followed by a decision of a clinician to treat with a full course of anti-tuberculosis chemotherapy. Diagnostic procedures including imageological methods, blood tests or laparotomies for excision biopsies, Mantoux tests, and BCG challenge tests were also used for the diagnosis of EPTB. In all the cases of EPTB, sputum examinations and chest radiographs were used to investigate the involvement of lung parenchyma. Our hospital lacks the facilities for culture and drug susceptibility testing.

### Statistical analysis

Data were analyzed using SPSS (Statistical Package for Social Sciences, version 12). Demographic variables, life-style factors and clinical characteristics were compared between EPTB and PTB groups. The Chi square test for categorical variables and the Mann-Whitney U test for continuous variables (e.g. age and contact-disease interval) were used to test for differences between the groups. Epi Info was used to carry out chi square for trend tests after stratification by gender. Our main analysis was carried out using unconditional logistic regression analysis, in which patients with EPTB were treated as cases and patients with PTB only were treated as controls. Multi-variate logistic regression analysis was used to identify the possible risk factors for EPTB. Odds ratios (OR), 95% confidence intervals (95% CI) and p-values were calculated for each potential predictor variable.

## Results

A total of 526 tuberculosis patients was registered in the DOTS clinic of MTH during the study period. Fifty two cases were not included due to incomplete recording of information (17) and missing medical case files (35). Four hundred and seventy four cases of TB were included in the final analysis. Two hundred and thirty patients (48.5%) were classified as EPTB and 244 (51.5%) as PTB. Of the 244 cases of PTB, 40 (16.4%) had hilar lymph node enlargement and/or pleural pathology (unilateral or bilateral). Of the 230 cases of EPTB, 38 (16.5%) had concurrent PTB. Demographic and descriptive data on the patients by TB type are shown in Table 1.

**Table 1:** Comparison of pulmonary and extra pulmonary tuberculosis according to demographic, life style and clinical characteristics

**Characteristics**	**EPTB (n = 230)**	**PTB (n = 244)**	**p-value**
**Demographic characteristics**			
< 25 years	94 (40.9%)	69 (28.3%)	< 0.001
25–50 years	86 (37.4%)	63 (25.8%)	
> 50 years	50 (21.7%)	112 (45.9%)	
Gender (Male/Female)	119/111	170/74	< 0.001
**Clinical characteristics**			
Presence of Diabetes mellitus	2 (0.9%)	16 (6.6%)	0.001
Use of immunosuppressive drugs	3 (1.3%)	20 (8.2%)	< 0.001
Past history of pulmonary tuberculosis	10 (4.3%)	37 (15.2%)	0.001
History of contact with TB patients	19 (8.3%)	28 (11.5%)	0.07
Median contact-disease interval in months (quartiles)	24 (6, 54)	24 (6, 84)	0.66
HIV positive patients* Number positive/Number tested	10/48 (20.1%)	6/31 (19.4%)	0.99
**Smoking habits**			
Never smoked	166 (72.2%)	113 (46.3%)	< 0.001
Currently smoking	34 (14.8%)	81 (33.2%)	
Quit at least 6 months previously	20 (8.7%)	45 (18.5%)	
**Alcohol use**			
Never drank alcohol	184 (80%)	160 (65.6%)	< 0.001
Currently drinking	24 (10.4%)	49 (20.1%)	
Quit at least 6 months previously	12 (5.2%)	30 (12.3%)	

* HIV status was not known for all the patients.

### Demographic factors

**Figure 1:**
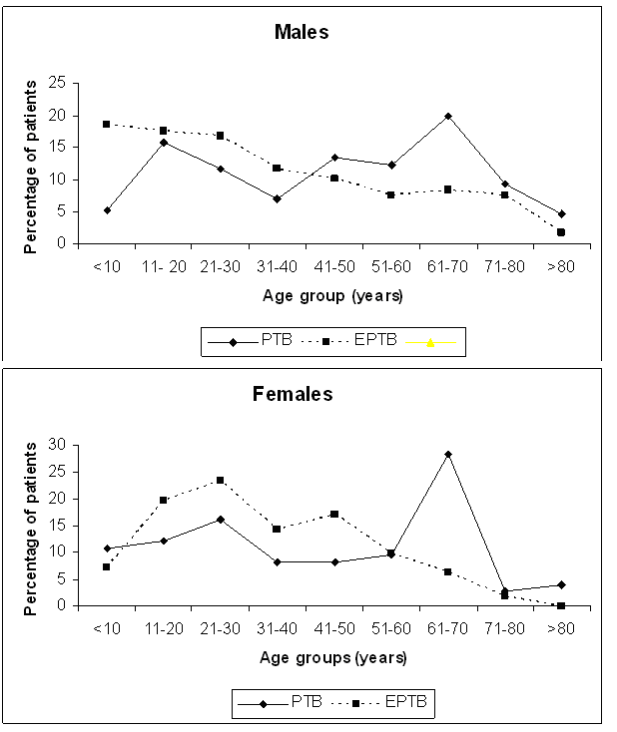
Proportional age distribution for EPTB and PTB among the 474 tuberculosis patients stratified by gender.

The overall male to female ratio of TB cases was 1.6 (289/185). For EPTB patients, the male to female ratio was 1.07 (119/111), but 2.29 (170/74) for PTB patients. The difference was statistically significant (p < 0.001). The median age of EPTB patients (29.5 years) was lower than that of PTB patients (47.5 years) (p < 0.001) (Table 1). Since the gender distributions were also different for PTB and EPTB, we examined age distributions after stratifying by gender and TB type. Figure 1 shows the age distributions of EPTB and PTB for males and females, confirming the tendency for EPTB to occur at younger ages (< 25 years) in both genders. The age at incidence of PTB showed evidence of a bimodal pattern, with peaks at 15–25 years and 60–70 years.

### Life-style factors

The proportion of 'ever smoked', (those who were either currently smoking or ex-smokers) was significantly higher among PTB patients (51.7%) compared to EPTB patients (23.5%). Similarly, a significantly higher proportion (i.e. 32.4%) of PTB patients was either currently consuming alcohol or former alcohol drinkers. (Table 1). In both sexes higher proportions of PTB patients had 'ever smoked' as compared to EPTB patients and this difference was statistically significant (p < 0.05).

### Clinical characteristics

As compared to EPTB patients a significantly higher proportion of PTB patients were suffering from diabetes mellitus (0.9% vs. 6.6%) (p < 0.001) or had a history of treatment with immunosuppressive drugs like steroids or anti-cancer drugs (1.3% vs. 8.2%) (p < 0.001). History of treatment for pulmonary TB (4.3% vs. 15.2%) in the past was also more common among PTB patients. The proportion of patients who had a history of contact with a known case of TB (8.3% vs. 11.5%) was higher among PTB patients but the duration from contact with a case of TB to onset of TB was virtually identical among PTB and EPTB groups. HIV status was known for only 79 out of 474 (16.6%) patients (Table 1), with about 20% positivity in both PTB and EPTB groups, providing no evidence of a difference in positivity rates between the two groups (p = 0.99). However, there may have been some selection bias in terms of who was tested for HIV, masking a difference in HIV positivity between the groups.

### Sites of EPTB

**Figure 2:**
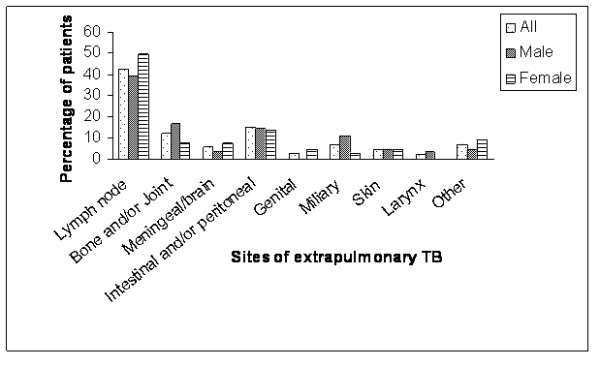
Distribution of sites of extra-pulmonary tuberculosis.

Sites of EPTB according to gender are illustrated in Figure 2. The most common site of EPTB was the lymph nodes (42.6%) followed by the peritoneum and/or intestines (14.8%), then bones and/or joints (12.4%). Other sites were miliary (7.2%), meninges/brain (7.2%), skin (4.8%), genital (2.9%) and larynx (2.4%). Fifteen EPTB patients (5.3%) had TB at other sites, namely the middle ear (4), disseminated (miliary) TB (4), pericardium (3), and the breast, thyroid, salivary gland and soft tissue (one each).

### Multivariate comparison of EPTB (cases) and PTB patients (controls)

The results of the multi-variate logistic regression analyses are presented in Table 2. The results suggest that younger age and female gender were associated with EPTB relative to PTB, whereas diabetes mellitus, use of immunosuppressive drugs, past history of PTB and having been a smoker are more likely to be associated with PTB than EPTB. The likelihood of development of EPTB was more than two-fold higher at younger ages (< 25 years) and one and half times higher among females than in males.

**Table 2:** Multi-variate logistic regression model determining the independent risk factors for development of extra pulmonary tuberculosis relative to pulmonary tuberculosis.

**Variables**	**P Value**	**Odds ratio**	**95% CI**
**Age (Years)**			
> 50		1	-
25–50	0.61	0.85	(0.46–1.58)
< 25	0.04	1.79	(1.04–3.09)
**Gender**			
Male	0.02	1	-
Female		1.67	(1.08–2.58)
**Diabetes mellitus**			
No	0.01	1	-
Yes		0.14	(0.03–0.65)
**Use of immunosuppressive drugs**			
No	0.01	1	-
Yes		0.14	(0.03–0.62)
**Past history of tuberculosis**			
No	0.001	1	-
Yes		0.25	(0.12–0.55)
Smoking habits*			
Never smoked		1	-
Currently smoking	0.001	0.34	(0.18–0.63)
Quit at least 6 months previously	0.08	0.45	(0.21–1.09)
Alcohol use*			
Never drank alcohol			
Currently drinking	0.68	1.21	(0.49–3.01)
Quit at least 6 months previously	0.56	1.34	(0.50–3.57)

* A person who quit smoking or drinking alcohol less than 6 months ago was coded as current smoker or currently drinking. Information about smoking and alcohol intake was not recorded in 15 case files. Therefore, 15 files were not included in the final regression analysis model.

## Discussion

Our study reports epidemiology and risk factors of EPTB from a high-burden country where the prevalence of HIV is low [13]. To our knowledge no similar study has been reported from other south Asian countries or other high TB burden countries. Younger age and female gender were strongly associated with EPTB, but smoking, diabetes mellitus, use of immunosuppressive drugs/steroids, and past history of pulmonary TB were associated with PTB.

### Sites of EPTB

In our study, the lymph nodes were the most common site of EPTB. Earlier studies have suggested that localization of EPTB may be variable. In Hong Kong [4], the genitourinary system and the skin were the common sites, whereas in the USA [3], bones and/or joints were the most common sites. Results of our study are comparable to two studies from Turkey [5,6] which reported that lymph nodes accounted for nearly half the cases of EPTB. In some studies clinical diagnosis of lymph node tuberculosis has been strengthened by a positive culture of *Mycobacterium tuberculosis *complex [3,6,11]. However, due to lack of facilities for culture in our setting, lymph node TB is usually diagnosed by fine needle aspiration cytology and/or node excision followed by histopathological examination for granulomatous inflammation with caseous necrosis. Therefore, we cannot rule out some mis-diagnosis of lymph node TB.

### Risk factors for EPTB

Earlier studies of risk factors for EPTB had a lower proportion of EPTB cases [3,11]. But in our study, proportions of PTB and EPTB were almost same, possibly because it was carried out in the main referral centre for the region and the proportion of EPTB may be higher due to availability of diagnostic facilities. Pulmonary TB cases are also diagnosed at primary health care centres because of the decentralization of diagnostic and treatment facilities under NTP. This means that they are often never referred to MTH. Gender differences observed in our study confirm the findings of previous studies in both developing [16,17] and developed countries [18,19]. This may be a consequence of gender differences in both exposures to TB infection and prevalence of susceptibility risk factors (e.g., smoking) [16]. Other possible factors accounting for the difference are stigma associated with having TB and lack of access to health care, especially for females, in some developing countries like Nepal [17].

The other main risk factor for EPTB relative to PTB that we identified was being younger than 25 years. This is consistent with studies from the USA [11] and Europe [12] which have reported that younger age was an independent risk factor for EPTB. Other studies from the USA [3] and Turkey [5], have reported that age was not associated with EPTB. These inconsistencies could be due to differences in prevalence of host-related factors or important co-exposures.

In our study, only 4.1% of the patients less than 25 years of age were "ever smokers" as compared to 74.8% of the patients who were aged 25 years or more. Proportions of "ever smokers" differed between males (46.8%) and females (27.1%). This raises the possibility that the age and sex differences between PTB cases (in whom smoking was more common) and EPTB cases could be a result of confounding by smoking. However, after adjusting for potential confounding factors (including smoking) by logistic regression analysis, younger age and female gender remained strongly associated with EPTB. Therefore, after primary infection in the lungs the probability of reactivation at an extra-pulmonary site may be higher at younger age. It would be useful to confirm the association of age and gender with EPTB in other high-burden countries. Our results suggest that at older ages reactivation of TB was common in the lungs. This may be due to decreased local immunity in the lungs in the elderly as a result of associated life-style factors (smoking) or co-morbid conditions which may predispose to re-activation in the lungs. A recent study from the UK has reported that co-morbid conditions like emphysema and bronchitis were independent risk factors for PTB [20].

In our study, smoking was associated with PTB. This is consistent with a meta-analysis which reported that smoking is a risk factor for TB infection and for pulmonary TB disease [21]. Another report has suggested that smoking is associated with relapse of TB and smokers are less likely to have isolated extrapulmonary TB [22]. We also found that past history of TB was associated with PTB, although we could not identify if this was as a result of reactivation (relapse) or reinfection [23]. However, evidence suggests that in high-burden countries reinfection is more common than relapse [24]. Therefore history of smoking and contact with a case of TB should arouse a high degree of suspicion for active TB. Such information would be useful for screening the patients for TB. Currently there are no health education campaigns or legislation regarding sales of cigarettes in Nepal. This is significant public health concern in view of tuberculosis control.

Our results of comparison of EPTB and PTB by logistic regression analysis suggested that factors like diabetes mellitus, use of immunosuppressive drugs/steroids and past history of TB are associated with PTB compared to EPTB. Our results are consistent with other studies that have reported an association between diabetes mellitus and PTB [25,26]. However, study from Turkey [5] examined the association of diabetes, use of immunosuppressive drugs/steroids and past history of TB with EPTB but found no association with any of these factors. A study from the UK [20] reported that use of immunosuppressive drugs/steroids and co-morbid conditions were associated with PTB. Therefore it is important to periodically screen the patients with chronic conditions like diabetes, those on immunosuppressive drugs/steroids for occurrence of tuberculosis.

Studies from developed countries have reported an increasing trend of EPTB among HIV infected persons [9,10] and HIV infection is associated with EPTB [3,10,12]. A recent study from a large tertiary hospital in south India reported that EPTB showed an increasing trend among HIV infected patients [12]. In Nepal, the adult HIV/AIDS prevalence is 0.5% [13]. Testing for HIV infection is carried out based on epidemiological and/or clinical suspicion and only a fraction of our cases were tested. Therefore, we did not have enough data to carry out a useful analysis of the association between HIV infection and EPTB. We carried out a logistic regression analyses excluding those cases which were HIV-positive. The results of our main logistic regression analyses did not change. The reported rate of HIV seropositivity among TB cases in Nepal was only 2.4% in the year 2001/02 [13]. The current rate may be higher, but probably not high enough to make a substantive difference to our conclusions. However it is important to screen the HIV/AIDS patients for TB despite the low incidence of HIV/AIDS in Nepal. Further studies are required to study the association between TB and HIV/AIDS.

Our study had several other limitations. Information about life style factors was sometimes incompletely recorded in the files. Therefore, we could not study the effect of amount and duration of smoking and alcohol use on EPTB. The PTB and EPTB patients were selected from a tertiary-care hospital. Hence, the cases we studied may not be representative of those occurring in the general population. Information about nutritional status and microbial factors was not available from the case files in our setting. A further limitation of the study was that it only identified factors that differed between PTB and EPTB. There may be some risk factors common to both forms of TB that could not be identified by our analysis.

## Conclusion

Results of our study suggest that younger age and female gender may be independent risk factors for EPTB, relative to PTB in Nepal. Whether this is common to other high-burden countries also is yet unclear. Further studies in other high burden countries are needed. In developing countries, the proportion of EPTB is relatively low and EPTB is less infectious than PTB. Therefore, EPTB is usually not prioritized for case finding strategies in TB control programmes. However, based on our results TB control programmes might usefully target young and female populations for early diagnosis of EPTB to decrease TB morbidity and mortality.

## Competing interests

The author(s) declare that they have no competing interests.

## Authors' contributions

CTS was the primary researcher, conceived the study, designed, participated in data collection, and drafted the manuscript for publication.

KVP assisted in conception, design, data collection and preparation of first draft of manuscript.

SCV designed and conducted data analysis and assisted in manuscript preparation.

HSJ designed data entry and analysis, and assisted in manuscript preparation

MNB interpreted the results, and reviewed the initial and final drafts of the manuscript.

All the authors read and approved the final manuscript for submission for publication.

## Pre-publication history

The pre-publication history for this paper can be accessed here:


